# Supplementary material for: The risk of indoor sports and culture events for the transmission of COVID-19
Source: Nat Commun. 2021 Aug 19;12:5096. doi: 10.1038/s41467-021-25317-9 (PMC8376924; doi:10.1038/s41467-021-25317-9)
Supplement: Supplementary file 1 — Supplementary Information [file 41467_2021_25317_MOESM1_ESM.pdf]

The Risk of Indoor Sports and Culture Events for the Transmission of COVID-19

Supplementary Information

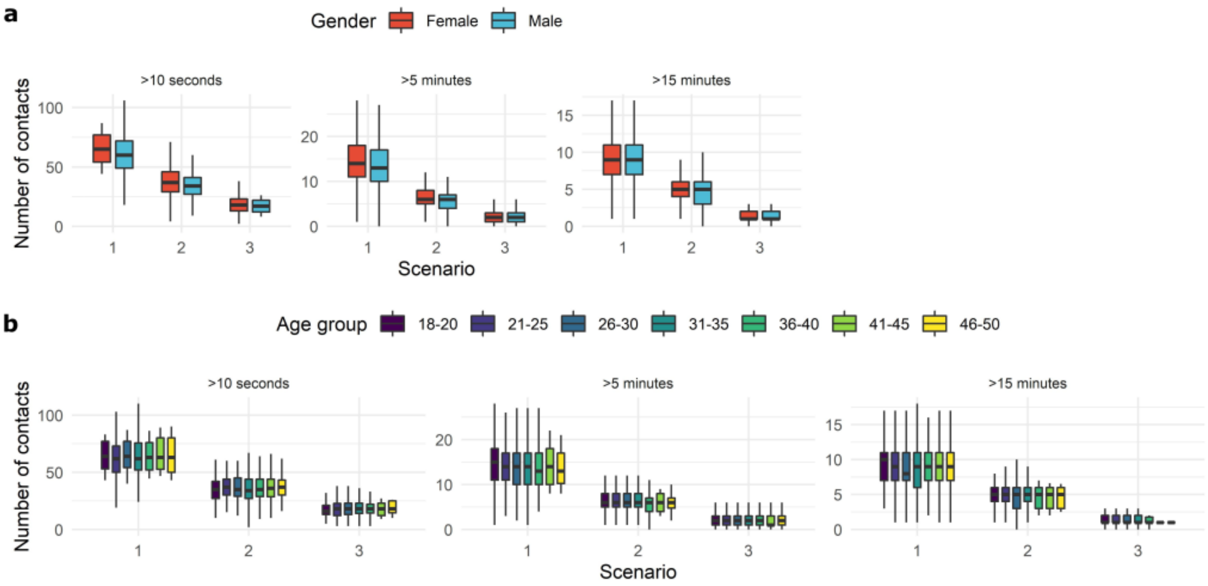

Supplementary Fig. 1: **Number of contacts in scenarios 1-3 by duration (>10 s, >5 min, >15 min) over all settings. a** by gender (red = female, blue = male) and **b** by age group (dark blue = 18-20 years, medium blue = 21-25 years, light blue = 26-30 years, green-blue = 31-35 years, dark green = 36-40 years, light green = 41-45 years, yellow = 46 – 50 years). The center line represents the median, the box limits the upper and lower quartiles and whiskers extend from the hinge to the smallest/largest value no further than 1.5 * IQR from the hinge. $n_{\text{Scenario 1}}$ = 1192 participants, $n_{\text{Scenario 2}}$ = 1158 participants, $n_{\text{Scenario 3}}$ = 1054 participants.

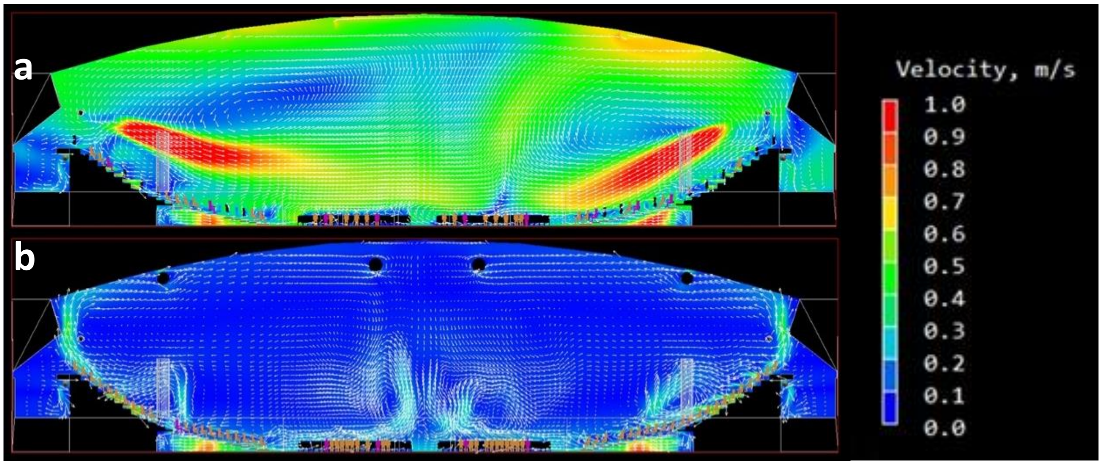

Supplementary Fig. 2: Vertical section of the Quarterback Immobilien Arena. a air velocities of ventilation version (VV) 1. Red areas: air velocities ≥ 1m/s; nozzles: 15.3 m/s. **b** air velocities of VV2. The stationary eddies emerging above the east and west grandstands are visible.

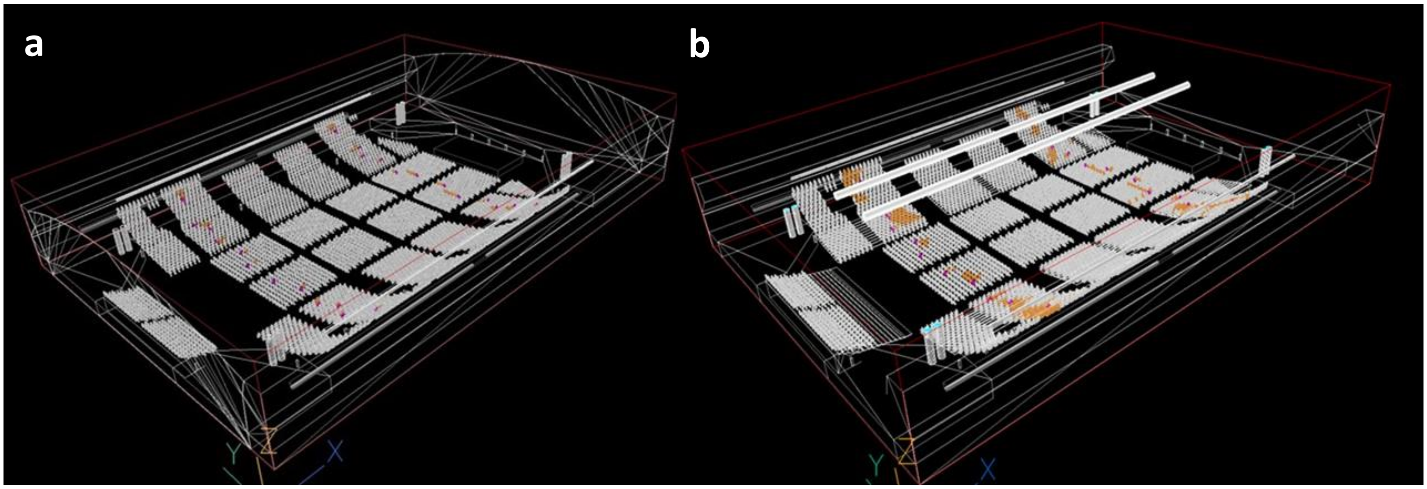

Supplementary Fig. 3: Overview of the Quarterback Immobilien Arena with the position of all 24 infectious individuals in red and respectively infected individuals in orange for a) ventilation version (VV) 1 and b) VV2.

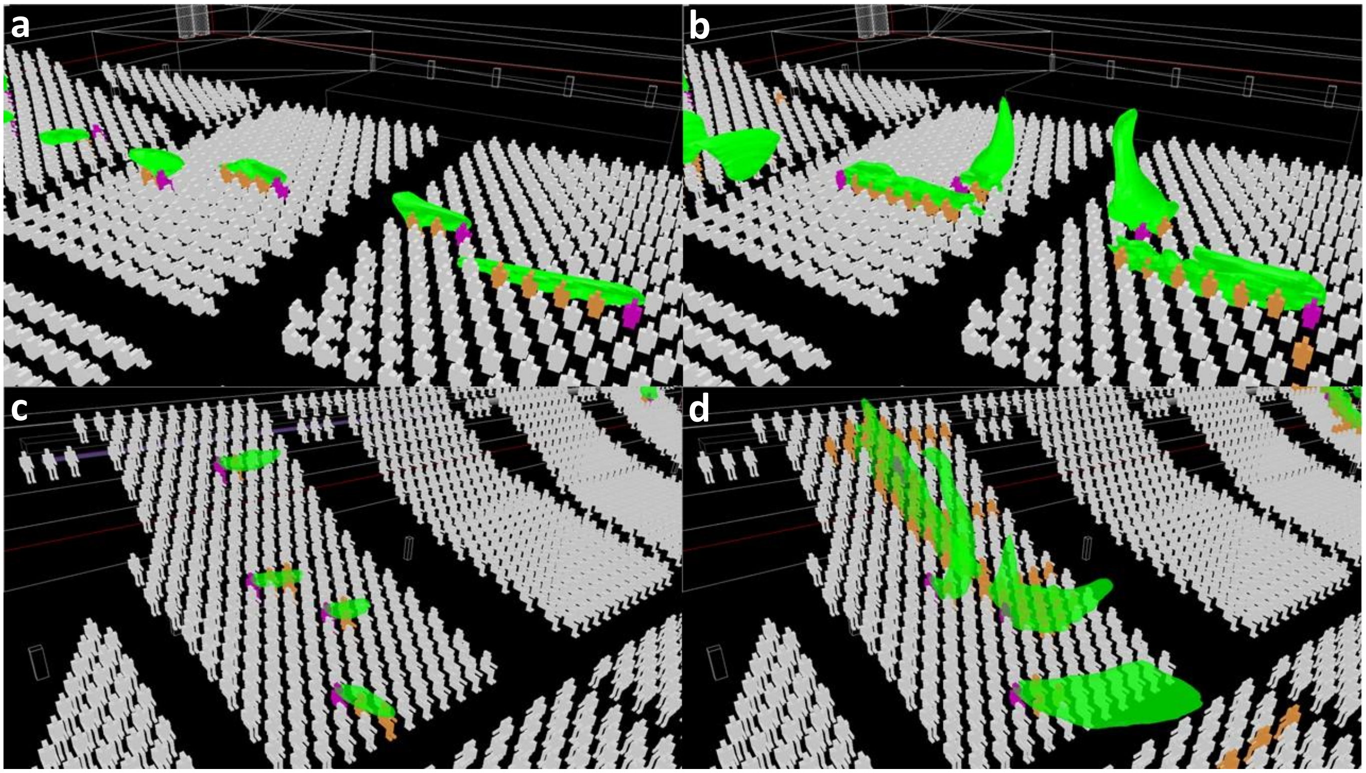

Supplementary Fig. 4: Aerosol concentrations displayed as isosurfaces (green) around the infectious individuals. Emitters/infectious individuals (violet), recipients/infected individuals (brown). **a** movement of the aerosols as isosurfaces from emitters to recipients in the stalls in front of the stage in ventilation version (VV) 1. **b** movement of the aerosols as isosurfaces from emitters to recipients in the stalls in front of the stage in VV2. **c** movement of the aerosols as isosurfaces from emitters to recipients on the west grandstand in VV1. **d** movement of the aerosols as isosurfaces from emitters to recipients on the west grandstand in VV2.

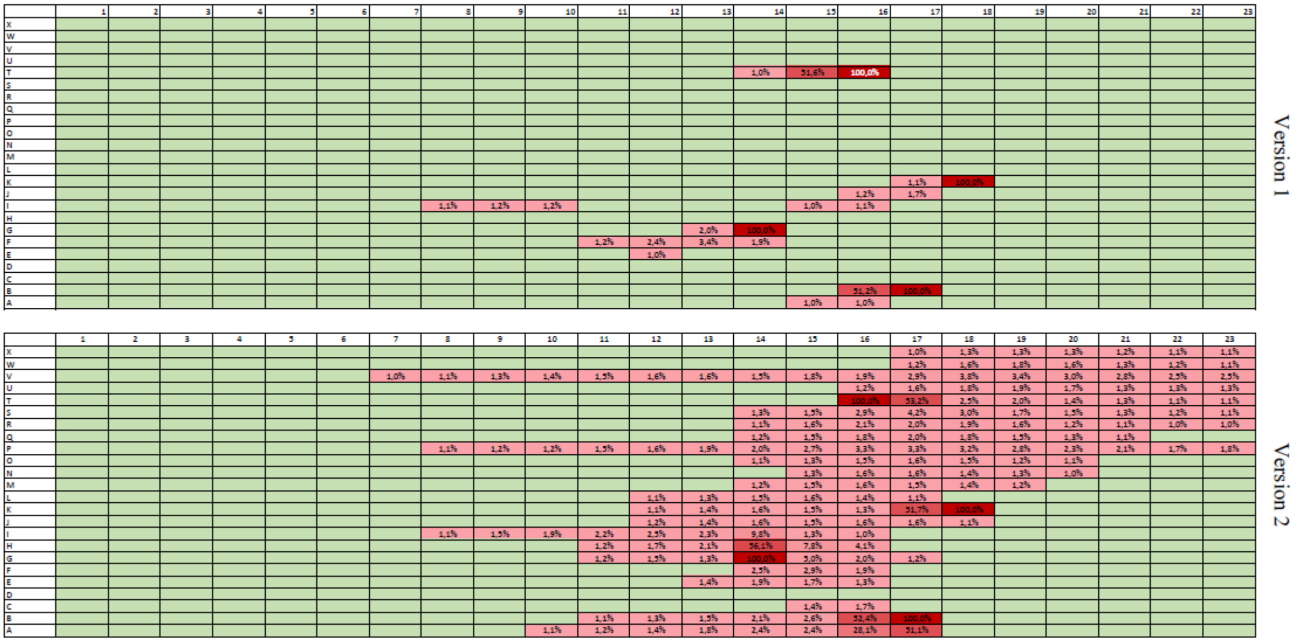

Supplementary Fig. 5: Two seating blocks (OF5, OM5) within the arena with amount of aerosol exposure (0.5-10µm) resulting from infectious individuals (indicated in dark red with black frame). People on green seats will receive <1% of the emitted aerosol amount. Red seats receive more than 1%. Current ventilation version 1 (upper part), simulated ventilation version 2 (lower part). The locations of these two areas are detailed in Extended Data Figure 8.

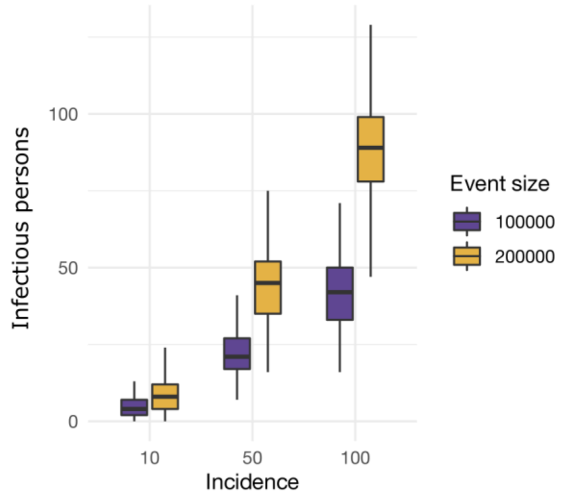

Supplementary Fig. 6: Average number of infectious persons in MGE by total number of people attending events per month (event size 100,000 people in purple, event size 200,000 people in yellow) and incidence in Scenario 1 with people using masks and bad ventilation. The center line represents the median, the box limits the upper and lower quartiles and whiskers extend from the hinge to the smallest/largest value no further than 1.5 * IQR from the hinge. $n_{\text{event size 1}}$ = 100,000, $n_{\text{event size 2}}$ = 200,000.

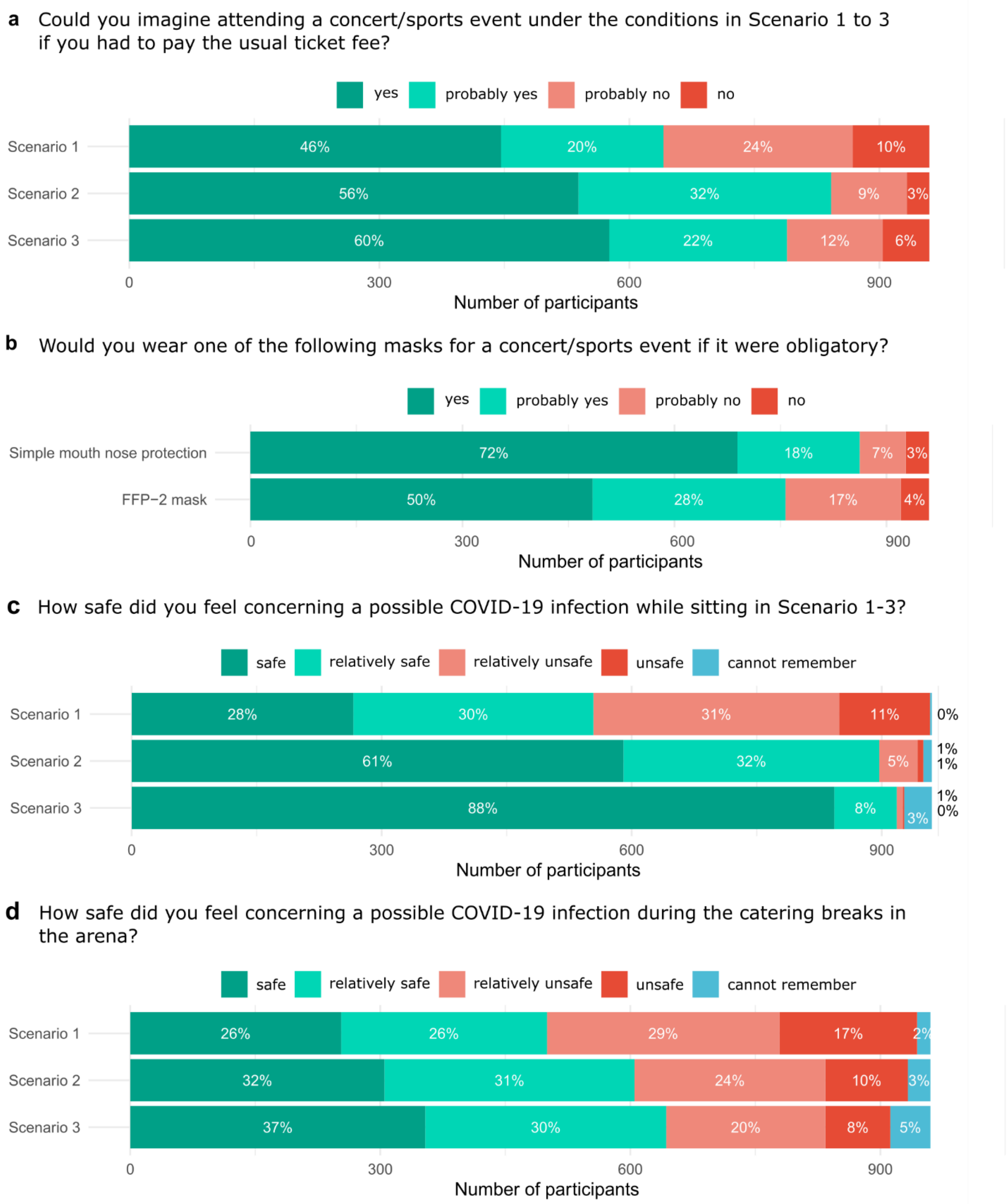

Supplementary Fig. 7: Results of the survey. n = 960 participants.

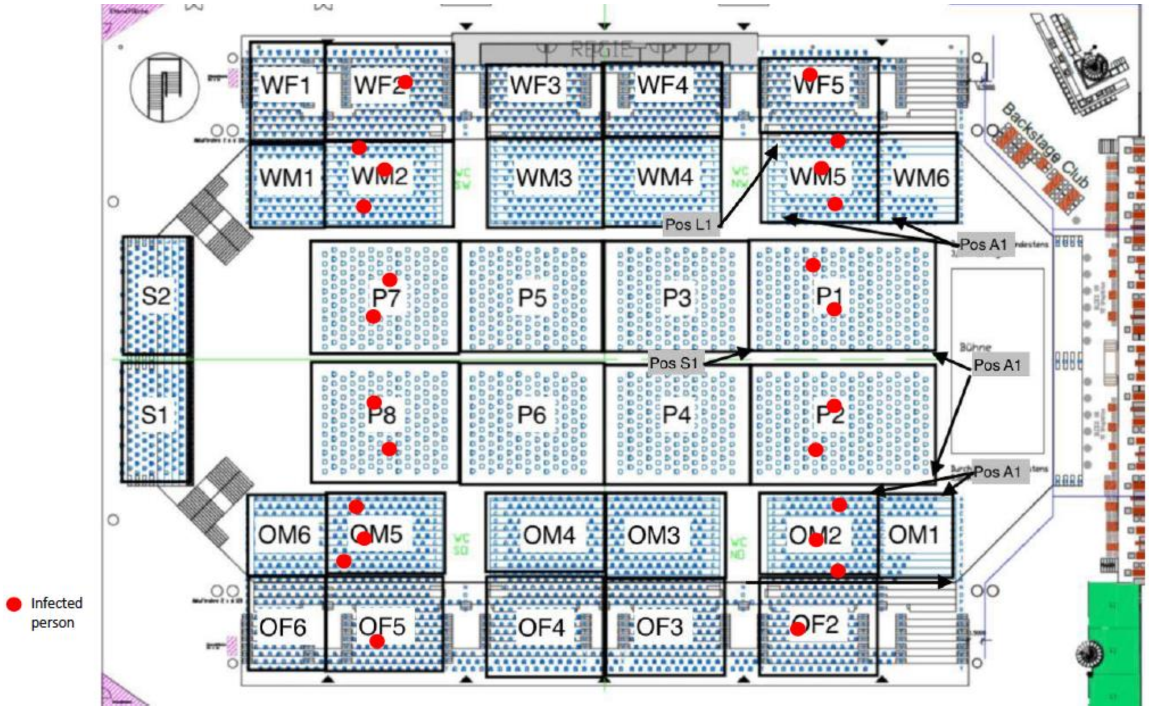

Supplementary Fig. 8: Distribution of infectious persons within the arena for the aerosol simulation. Red dots indicate infectious persons. WF: upper west grandstand; WM: lower west grandstand; OF: upper east grandstand; OM: lower east grandstand; P: floor

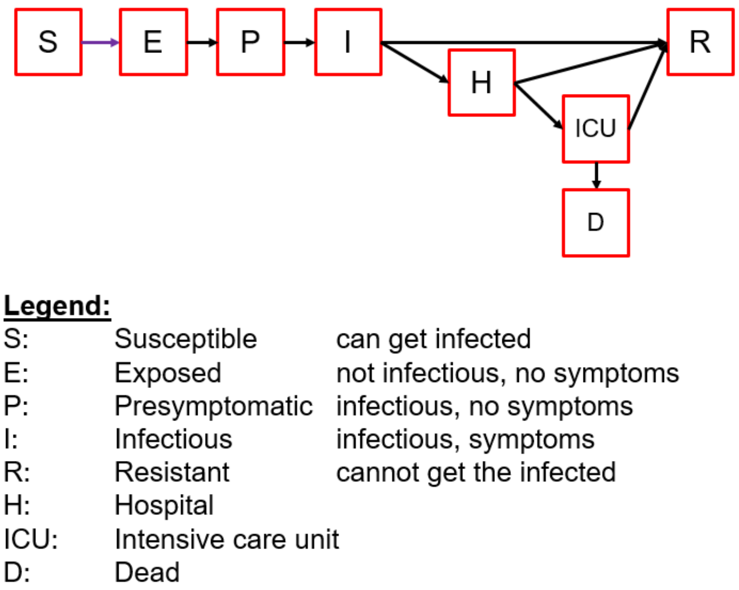

Supplementary Fig. 9: Extended SEIR model.

Supplementary Table 1. Socio-demographic characteristics of the participants in the live event (n = 1212).

**Variables**		**N (%)**
Age	18-25	355 (29.3)
	26-30	169 (13.9)
	31-35	248 (20.5)
	36-40	186 (15.3)
	41-45	143 (11.8)
	46-50	111 (9.2)
Gender	Male	443 (36.5)
	Female	767 (63.3)
	Other	2 (0.2)
Area of residence	Leipzig	471 (38.9)
	Saxony (outside Leipzig)	404 (33.3)
	Outside Saxony	337 (27.8)

Supplementary Table 2. Mean number of contacts of all participants, measured using mobile contact tracing devices.

Time cut-off	S	Mean number of measured contacts (± SD)					
		Total	Entry	1st half	half time	2nd half	Exit
≥ 10s	1	63.9 (±17.1)	30.8 (±12.1)	7.0 (±2.8)	24.5 (±9.2)	6.4 (±2.7)	15.3 (±6.7)
	2	36.4 (±12.0)	13.5 (±5.3)	3.7 (±1.5)	14.7 (±6.7)	4.3 (±1.8)	14.9 (±7.6)
	3	18.0 (±7.2)	6.1 (±3.5)	1.3 (±0.6)	8.3 (±5.1)	1.8 (±1.5)	6.1 (±3.6)
≥ 5min	1	14.1 (±5.2)	8.7 (±4.1)	5.3 (±2.3)	3.1 (±2.4)	4.4 (±2.0)	1.6 (±1.4)
	2	6.1 (±2.4)	4.9 (±2.1)	2.7 (±1.3)	2.6 (±1.5)	3.2 (±1.5)	1.1 (±0.9)
	3	2.2 (±1.5)	2.0 (±1.3)	1.0 (±0.3)	1.2 (±1.9)	1.0 (±0.7)	0.7 (±0.7)
≥ 15min	1	8.9 (±3.5)	5.1 (±2.5)	4.5 (±2.1)	1.8 (±1.3)	3.9 (±1.9)	0 (±0)
	2	4.7 (±1.9)	3.7 (±1.6)	2.3 (±1.2)	1.9 (±1.2)	2.9 (±1.4)	0 (±0)
	3	1.3 (±0.9)	1.1 (±0.6)	1.0 (±0.3)	0.8 (±0.7)	0.9 (±0.6)	0 (±0)
≥ 10s cumulative	1		30.8	32.8	52.1	54.1	63.9
	2		13.5	13.9	24.7	25.3	36.4
	3		6.1	6.3	13.3	14.1	18.0
≥ 5min cumulative	1		8.7	11.0	12.3	13.7	14.1
	2		4.9	5.2	5.6	6.0	6.1
	3		2.0	2.0	2.1	2.2	2.2
≥ 15min cumulative	1		5.1	7.3	7.5	8.9	8.9
	2		3.7	4.0	4.2	4.7	4.7
	3		1.1	1.1	1.3	1.3	1.3

The mean number of total contacts longer than 10 seconds, 5 minutes and 15 minutes are shown for all participants, as well as contacts stratified by setting (entry, 1st half, half time, 2nd half, exit) within all scenarios (1, 2, and 3). Contacts can be counted more than once, depending on which setting they occur in, but are counted once in total. Cumulative data is provided by adding additional contacts within a setting to the previous number of contacts. SD = standard deviation, S = scenario.

Supplementary Table 3. Components of the hygiene practices in the three different scenarios.

	**Scenario 1**	**Scenario 2**	**Scenario 3**
**Seating**	No seats free	Every 2nd seat free<br>“Checkboard pattern”	Pairwise,<br>1.5 m circumference free
**Quadrants**	No	Yes	Yes
**Entrances**	2	4	8
**Catering**	Unrestricted	In quadrants	In quadrants
**Toilets**	Unrestricted	Every 2nd urinal closed	Every 2nd urinal closed

Supplementary Table 4. Number of exposed individuals by direct contact (≤1.5m) and through aerosol exposure for each ventilation version (VV). SD = standard deviation.

**Type of exposure**	**Mean number of exposed per each infected individual (±SD) in VV 1**			**Mean number of exposed per infected individuals (± SD) in VV 2**		
	**Scenario 1**	**Scenario 2**	**Scenario 3**	**Scenario 1**	**Scenario 2**	**Scenario 3**
**Direct contact**	9.0 (±3.5)	4.7 (±1.9)	1.3 (±0.9)	9.0 (±3.5)	4.7 (±1.9)	1.3 (±0.9)
**Aerosols**	3.5 (±2.9)	1.9 (±1.5)	0.7 (±1.0)	25.5 (±27.8)	11.8 (±13.5)	5.3 (±6.4)
**Total**	12.5	6.6	2.0	34.5	16.5	6.6

Supplementary Table 5. Parameters and equations used for the CFD Model.

**Parameters and equations**	**Details**
n	Number of particles in the respiratory air per second
$\rho_p = 1300$	Density of the aerosol in kg/m^3
$V_p = 4/3*\pi*(D/2)^3$	Particle volume in m^3
$V = n*V_p$	Entire volume flow particle type
$m = V* \rho_p$	Mass flow particle type
$T = 31.5°C$	Breathing temperature
$P1 = 100.13$	Air pressure in kPa
$rh = 0.5$	Relative humidity 50%
$V_{CO2} = 0.020 / 3600$	= 20 litre/h CO_2 in m3/s
Lung Volume = 4.5*1.8	= 12 breaths/minute = 8.1 litre/min
$V_{breath} = 0.0081 / 60$	= 0.000135 m^3/s
$\rho_{breath} = \rho(AirH_2O; T = T; R = rh; P = P1) = 1.135$	Density of respiratory air in kg/m3
$m_{breath} = V_{breath}*\rho_{breath} = 0.0001532$	Mass flow of breath in kg/s
$\rho_{CO2} = \rho(CO_2; T = T; P = P1) = 1.74$	Density of CO_2 in kg/m3
$m_{CO2} = VCO_2*\rho CO_2$	= 0.000009665 kg/s
$m_{sum} = 5.27*10^{-9}$	Sum (Column 4), aerosols in kg/s
$m_{total} = m_{sum} + m_{breath} + m_{CO2} = 0,0001629$	Total mass flow in kg/s
$Ai = m / m_{total}$	Amount of mass flow
$A_{breath} = m_{breath} / m_{total} = 0,9406$	Amount respiratory air
$A_{CO2} = m_{CO2} / m_{total} = 0.05933$	Amount CO_2
$A_{control} = A_{breath} + A_{CO2} + 32.4*10^{-6} = 1.0$	Sum (Column 5) $Ai = 32.4*10^{-6}$

Supplementary Table 6. Particle distribution used for the aerosol model.

**Run**	***D*** **[m]**	***n*** **[1/s]**	***V*** **[m^3/s]**	***m*** **[kg/s]**
1	5.000E-07	5500	3.600E-16	4.680E-13
2	5.000E-06	3500	2.291E-13	2.978E-10
3	1.000E-05	1000	5.236E-13	6.807E-10
4	3.000E-05	500	7.069E-12	9.189E-9
5	5.000E-05	500	3.272E-11	4.254E-08

D = diameter of the aerosols; *n* = number of particles in the respiratory air per second; *V* = particle volume; *m* = mass flow

Supplementary Table 7. Parameters for transmission model.

From	To	Assumed mean value in days (n; probability)	Details	Reference
E	P	3 (4; 0.5)	Latent period ranged from 2 to 4 days	[1,2]
P	I	1.5 (2; 0.25)	Duration of asymptomatic infectiousness ranged from 1 to 3 days	[3,4]
I	H	4 (6; 0.5)	Duration from having symptoms to being hospitalized was 4 days	[5]
H	ICU	2 (2; 0.5)	Duration from hospitalization to ICU ranged from 1 to 3.5 days	[1,2]
ICU	D	8 (10; 0.7)	Time from ICU to death ranged from 3.5 to 14 days	[1]
I	R	6 (10; 0.5)	5 to 10 incl. pre-symptomatic	[6]
H	R	11.8 (12; 0.9)	Duration from hospital admission to recovery ranged from 10 to 14 days (for non ICU-patients)	[7]
ICU	R	12 (22; 0.5)	Duration from ICU to recovery ranged from 8 to 17 days	[1,7]

E=exposed individuals (not infectious, no symptoms), P=pre-symptomatic individuals (infectious, no symptoms), I=infectious individuals (infectious, symptoms), H=hospital submission, ICU=submission to intensive care unit, D=death, R=resistant individuals.

Supplementary Table 8. Percentage progressing from one state to another, divided into age groups and individuals modelled per age group.

Age group	Percentage with no symptoms (E→R)	Percentage hospitalized (I→H)	Percentage in ICU per hospitalized (H→ICU)	Lethality (ICU→D)	Number of individuals modelled [8]
0-4	17% for all age groups	10.0%	20.84%	6.60%	31497
5-9		10.0%	9.32%	6.60%	27905
10-14		10.0%	9.10%	5.0%	23801
15-19		10.0%	6.77%	5.0%	24831
20-24		10.0%	7.02%	10.0%	42208
25-29		10.0%	14.18%	10.0%	48165
30-34		12.0%	14.81%	25.0%	59903
35-39		12.0%	8.89%	25.0%	49607
40-44		15.0%	13.86%	25.0%	38700
45-49		15.0%	17.24%	25.0%	33017
50-54		21.0%	11.55%	62.73%	36880
55-59		21.0%	19.13%	62.73%	37173
60-64		27.0%	22.85%	59.09%	30703
65-69		27.0%	38.93%	59.09%	30181
70-74		39.0%	30.28%	80.0%	21741
75-79		39.0%	45.10%	80.0%	29420
80-84		53.0%	35.55%	80.0%	23872
85-++		53.0%	28.22%	80.0%	17355
Ref	[9]	Adjusted to fit to hospitalization rate in Schleswig-Holstein [10]	Adjusted/multiplied to fit to the total number of ICU admissions in Germany [11]	Adjusted to fit to mortality rate in Germany [12]	

Supplementary Table 9. Number of contacts per household size, setting and age group.

Age group	Type of contact														
	Household size of 1			Household size of 2			Household size of 3			Household size of 4			Household size of 5		
	House-hold	School/work	Other	House-hold	School/work	Other	House-hold	School/work	Other	House-hold	School/work	Other	House-hold	School/work	Other
0-4	0.93	1.44	1.19	1.15	1.23	1.19	1.41	1.05	1.19	1.73	0.90	1.19	2.12	0.77	1.19
5-9	0.93	1.65	1.35	1.15	1.41	1.35	1.41	1.20	1.35	1.73	1.02	1.35	2.12	0.87	1.35
10-14	0.93	1.88	1.53	1.15	1.61	1.53	1.41	1.37	1.53	1.73	1.17	1.53	2.12	1.00	1.53
15-19	0.73	2.15	1.74	0.93	1.83	1.74	1.20	1.56	1.74	1.53	1.34	1.74	1.96	1.14	1.74
20-24	0.73	1.12	1.78	0.93	1.12	1.78	1.20	1.12	1.78	1.53	1.12	1.78	1.96	1.12	1.78
25-29	0.73	1.12	1.65	0.93	1.12	1.65	1.20	1.12	1.65	1.53	1.12	1.65	1.96	1.12	1.65
30-34	0.73	1.12	1.53	0.93	1.12	1.53	1.20	1.12	1.53	1.53	1.12	1.53	1.96	1.12	1.53
35-39	0.73	1.12	1.42	0.93	1.12	1.42	1.20	1.12	1.42	1.53	1.12	1.42	1.96	1.12	1.42
40-44	0.73	1.12	1.32	0.93	1.12	1.32	1.20	1.12	1.32	1.53	1.12	1.32	1.96	1.12	1.32
45-49	0.73	1.12	1.22	0.93	1.12	1.22	1.20	1.12	1.22	1.53	1.12	1.22	1.96	1.12	1.22
50-54	0.73	1.12	1.13	0.93	1.12	1.13	1.20	1.12	1.13	1.53	1.12	1.13	1.96	1.12	1.13
55-59	0.73	1.12	1.05	0.93	1.12	1.05	1.20	1.12	1.05	1.53	1.12	1.05	1.96	1.12	1.05
60-64	0.73	0.15	0.98	0.93	0.15	0.98	1.20	0.15	0.98	1.53	0.15	0.98	1.96	0.15	0.98
65-69	0.73	0.15	0.91	0.93	0.15	0.91	1.20	0.15	0.91	1.53	0.15	0.91	1.96	0.15	0.91
70-74	0.73	0.00	0.84	0.93	0.00	0.84	1.20	0.00	0.84	1.53	0.00	0.84	1.96	0.00	0.84
75-79	0.73	0.00	0.78	0.93	0.00	0.78	1.20	0.00	0.78	1.53	0.00	0.78	1.96	0.00	0.78
80-84	0.73	0.00	0.72	0.93	0.00	0.72	1.20	0.00	0.72	1.53	0.00	0.72	1.96	0.00	0.72
85++	0.73	0.00	0.67	0.93	0.00	0.67	1.20	0.00	0.67	1.53	0.00	0.67	1.96	0.00	0.67

Supplementary References

1. an der Heiden, M. & Buchholz, U. Modellierung von Beispielszenarien der SARS-CoV-2-Epidemie 2020 in Deutschland. (2020) doi:10.25646/6571.2.
2. Khailaie, S. *et al.* Development of the reproduction number from coronavirus SARS-CoV-2 case data in Germany and implications for political measures. *BMC Med.* **19**, 1–16 (2021).
3. Böhmer, M. M. *et al.* Investigation of a COVID-19 outbreak in Germany resulting from a single travel-associated primary case: a case series. *Lancet Infect. Dis.* **20**, 920–928 (2020).
4. Xia, W. *et al.* Transmission of corona virus disease 2019 during the incubation period may lead to a quarantine loophole. (2020) doi:10.1101/2020.03.06.20031955.
5. Docherty, A. B. *et al.* Features of 20 133 UK patients in hospital with covid-19 using the ISARIC WHO Clinical Characterisation Protocol: Prospective observational cohort study. *BMJ* **369**, 1–12 (2020).
6. Wölfel, R. *et al.* Virological assessment of hospitalized patients with COVID-2019. *Nature* **581**, 465–469 (2020).
7. Wang, D. *et al.* Clinical Characteristics of 138 Hospitalized Patients with 2019 Novel Coronavirus-Infected Pneumonia in Wuhan, China. *JAMA - J. Am. Med. Assoc.* **323**, 1061–1069 (2020).
8. Stadt Leipzig (2020). Bevölkerungsbestand. Available at: https://statistik.leipzig.de/statcity/table.aspx?cat=2&rub=5&obj=0 (Accessed: 15 May 2021).
9. Byambasuren, O. *et al.* Estimating the extent of asymptomatic COVID-19 and its potential for community transmission: Systematic review and meta-analysis. *J. Assoc. Med. Microbiol. Infect. Dis. Canada* **5**, 223–234 (2020).
10. Landesportal Schleswig-Holstein (2021). Für Schleswig-Holstein gemeldete Corona-Fälle. Available at: https://www.schleswig-holstein.de/DE/Schwerpunkte/Coronavirus/Zahlen/zahlen_node.html (Accessed: 15 May 2021).
11. Flaxman, S. *et al.* Estimating the effects of non-pharmaceutical interventions on COVID-19 in Europe. *Nature* **584**, 257–261 (2020).
12. Robert-Koch-Institut (RKI) (2020). COVID-19 Datenhub. Available at: https://npgeo-corona-npgeo-de.hub.arcgis.com/search (Accessed: 15 May 2021).
