# Supplementary material for: The risk of indoor sports and culture events for the transmission of COVID-19
Source: Nat Commun. 2021 Aug 19;12:5096. doi: 10.1038/s41467-021-25317-9 (PMC8376924; doi:10.1038/s41467-021-25317-9)
Supplement: Supplementary file 3 — Reporting Summary [file 41467_2021_25317_MOESM3_ESM.pdf]

nature research

Corresponding author(s): Stefan Moritz
Rafael Mikolajczyk

Last updated by author(s): Jul 29, 2021

Reporting Summary

Nature Research wishes to improve the reproducibility of the work that we publish. This form provides structure for consistency and transparency in reporting. For further information on Nature Research policies, see our Editorial Policies and the Editorial Policy Checklist.

Statistics

For all statistical analyses, confirm that the following items are present in the figure legend, table legend, main text, or Methods section.

n/a	Confirmed	
[x]	[ ]	The exact sample size (*n*) for each experimental group/condition, given as a discrete number and unit of measurement
[x]	[ ]	A statement on whether measurements were taken from distinct samples or whether the same sample was measured repeatedly
[x]	[ ]	The statistical test(s) used AND whether they are one- or two-sided<br>*Only common tests should be described solely by name; describe more complex techniques in the Methods section.*
[ ]	[x]	A description of all covariates tested
[x]	[ ]	A description of any assumptions or corrections, such as tests of normality and adjustment for multiple comparisons
[ ]	[x]	A full description of the statistical parameters including central tendency (e.g. means) or other basic estimates (e.g. regression coefficient) AND variation (e.g. standard deviation) or associated estimates of uncertainty (e.g. confidence intervals)
[x]	[ ]	For null hypothesis testing, the test statistic (e.g. *F*, *t*, *r*) with confidence intervals, effect sizes, degrees of freedom and *P* value noted<br>*Give P values as exact values whenever suitable.*
[x]	[ ]	For Bayesian analysis, information on the choice of priors and Markov chain Monte Carlo settings
[x]	[ ]	For hierarchical and complex designs, identification of the appropriate level for tests and full reporting of outcomes
[x]	[ ]	Estimates of effect sizes (e.g. Cohen's *d*, Pearson's *r*), indicating how they were calculated

Our web collection on statistics for biologists contains articles on many of the points above.

Software and code

Policy information about availability of computer code

Data collection	No software was used for data collection.
Data analysis	The fluid dynamics calculations were conducted with the software PHOENICS (Version 2020, CHAM, London, United Kingdom) using the add-ons FLAIR and GENTRA (both Version 2020, CHAM, London, United Kingdom). Contact analysis and model construction were performed with R (version 4.0.2). Codes are available at http://doi.org/10.5281/zenodo.4647830 and http://doi.org/10.5281/zenodo.4770064

For manuscripts utilizing custom algorithms or software that are central to the research but not yet described in published literature, software must be made available to editors and reviewers. We strongly encourage code deposition in a community repository (e.g. GitHub). See the Nature Research guidelines for submitting code & software for further information.

Data

Policy information about availability of data

All manuscripts must include a data availability statement. This statement should provide the following information, where applicable:
- Accession codes, unique identifiers, or web links for publicly available datasets
- A list of figures that have associated raw data
- A description of any restrictions on data availability

Most of the quantitative results are provided in the supplemental tables. Additionally, data are publicly available at https://doi.org/10.5281/zenodo.5137667

Field-specific reporting

Please select the one below that is the best fit for your research. If you are not sure, read the appropriate sections before making your selection.

☒ Life sciences ☐ Behavioural & social sciences ☐ Ecological, evolutionary & environmental sciences

For a reference copy of the document with all sections, see nature.com/documents/nr-reporting-summary-flat.pdf

Life sciences study design

All studies must disclose on these points even when the disclosure is negative.

Sample size	Sample size calculations were not performed. We planned to include 4000 participants corresponding to half of the capacity of the arena and reflecting the mean event size for sports and culture events of the year 2019 at this location. Due to various reasons explained in the manuscript, 1212 persons took part in the experiment. We were aware of this issue before the study days and thus able to adapt the study design to a smaller number of participants by artificially decreasing the size of the event location by one fourth.
Data exclusions	No data was excluded.
Replication	Due to immense financial and organisatorial effort, there was only one study days and no replications. However, the different scenarios measured during the study day can be seen as internal validation of the data.
Randomization	Randomization was not necessary because all participants attended all szenarios and the different scenarios served as internal controls.
Blinding	Blinding was not possible during the event because, in order to implement hygiene concepts, participants had to be aware of them. The collected data were anonymized afterwards.

Reporting for specific materials, systems and methods

We require information from authors about some types of materials, experimental systems and methods used in many studies. Here, indicate whether each material, system or method listed is relevant to your study. If you are not sure if a list item applies to your research, read the appropriate section before selecting a response.

Materials & experimental systems

n/a	Involved in the study	
☒	☐	Antibodies
☒	☐	Eukaryotic cell lines
☒	☐	Palaeontology and archaeology
☒	☐	Animals and other organisms
☐	☒	Human research participants
☒	☐	Clinical data
☒	☐	Dual use research of concern

Methods

n/a	Involved in the study	
☒	☐	ChIP-seq
☒	☐	Flow cytometry
☒	☐	MRI-based neuroimaging

Human research participants

Policy information about studies involving human research participants

Population characteristics	Of the study population, 36.5% were male and 63.3% female. Furthermore, 29.3% were 18 to 25 years old, 13.9% between 26 and 30 years, 20.5% between 31 and 35 years, 15.3% between 36 and 40 years, 11.8% between 41 and 45 years and 9.2% between 46 and 50 years of age. Of the study participants, 38.9% were from Leipzig, 33.3% from the federal state of saxony and 27.8% from outside saxony.
Recruitment	Participants were invited through extensive media campaign to register voluntarily and free of charge via the study webpage (www.restart19.de) where comprehensive information about the event, its objectives, and risks were provided. Participants did not receive any kind of allowance, but food and drinks were free throughout the day. Our participants were not representive of the general population (most likely fans of the musician or people highly interested in science were recruite, thus creating bias). Furthermore, our participants were highly compliant possibly leading to bias in a way that contacts were lower than in a less compliant population.
Ethics oversight	The Ethics Committee of the Martin Luther University (Halle, Germany) approved the data collection for the RESTART-19 study. The responsible authorities of the Federal State of Saxony (Saechsisches Staatsministerium fuer Soziales und gesellschaftlichen Zusammenhalt) provided special permission for the mass event. The Public Health Authority of the city of Leipzig approved the hygiene and safety practice for the event date.

Note that full information on the approval of the study protocol must also be provided in the manuscript.
