# Supplementary material for: The risk of indoor sports and culture events for the transmission of COVID-19
Source: Nat Commun. 2021 Aug 19;12:5096. doi: 10.1038/s41467-021-25317-9 (PMC8376924; doi:10.1038/s41467-021-25317-9)
Supplement: Supplementary file 4 — Description of Additional Supplementary Files [file 41467_2021_25317_MOESM4_ESM.pdf]

Description of Additional Supplementary Files

Supplementary Data 1. Epidemiological outcomes from individual-based model

Supplementary Data 2. Increase of positive cases in percent based on data from individual-based model

Supplementary Movie 1. Simulation of particle flow within the arena in Ventilation Version 1
